# Resilience and Vulnerability to Pain and Inflammation in the Hippocampus

**DOI:** 10.3390/ijms18040739

**Published:** 2017-03-31

**Authors:** Verica Vasic, Mirko H. H. Schmidt

**Affiliations:** Molecular Signal Transduction Laboratories, Institute for Microscopic Anatomy and Neurobiology, Focus Program Translational Neuroscience (FTN), Rhine Main Neuroscience Network (rmn^2^), Johannes Gutenberg University, School of Medicine, Mainz 55131, Germany; verica.vasic@unimedizin-mainz.de

**Keywords:** neurogenesis, resilience, inflammation, pain, neuropsychiatric disorders

## Abstract

Increasing evidence demonstrates the importance of hippocampal neurogenesis, a fundamental mechanism of neuroplasticity associated with cognition and emotion, in correlation to neurodegenerative and psychiatric disorders. Neuropsychiatric disorders are often a result of chronic stress or pain followed by inflammation; all these conditions manifest cognitive deficits and impairments in neurogenesis. However, while some individuals are more susceptible to stress, others are able to adapt to new environments via mechanisms of resilience. In light of this emerging field and based on extensive research, the role of neurogenesis is summarized and presented as a potentially powerful therapeutic tool.

## 1. Introduction

The hippocampus is one of the brain privileged sites which host the process of neurogenesis—the generation of new neurons from neural stem cells in the adult brain of various species. Neurogenesis in the dentate gyrus of the hippocampus is associated with cognitive [1,2,3] and emotional [4,5] functions. These functions are shown to be impaired in normal aging, age-related neurodegenerative diseases, as well as neuropsychiatric diseases, and therefore they are increasingly examined through the prism of neurogenesis [6,7,8]. Neuropsychiatric disorders are predicted to be the second highest cause of global disease burden by the year 2030 [9,10]. A major risk factor for developing depression and mental disorders is stress, particularly chronic stress, associated with inflammation. Moreover, there is significant overlap between depression and other conditions, such as chronic pain, anxiety, and neurodegeneration [11], all sharing similar symptoms, functional dysregulations, and molecular mechanisms; therefore, they should be studied in close connection. Alternatively, even upon stress and trauma, some individuals do not develop disease but manage to adapt to the new environment and maintain homeostasis. These individuals employ mechanisms of resilience. Recent data show that hippocampal neurogenesis may play a role in responses to stress, although it is not yet fully clear under which circumstances neurogenesis promotes resilience or susceptibility to stress. Thus, it is important to examine how neurogenesis can contribute to stress resilience or vulnerability, as it may enable the development of effective treatments for neuropsychiatric disorders.

Exposure to stress activates the hypothalamic-pituitary-adrenal (HPA) axis in a three-step process: corticotropin-releasing hormone (CRH) is released from the paraventricular nucleus (PVN) of the hypothalamus, leading to the secretion of adrenocorticotropic hormone (ACTH) from the pituitary gland, which finally results in the release of the glucocorticoids corticosterone and cortisol into the blood [12]. These glucocorticoids, cortisol in humans and corticosterone in rodents [13], feedback onto two types of receptors in the brain: mineralocorticoid receptors (MRs) and glucocorticoid receptors (GRs), which are both highly expressed in limbic structures of the brain, including the hippocampus [14], thus making it susceptible to stress. The HPA axis is also involved in the regulation of inflammation and chronic stress [15]; their role will be further discussed in the context of neurogenesis and neuropsychiatric disorders.

## 2. Hippocampus

### 2.1. Structure and Function

The hippocampus, folded into the medial temporal lobe with a distinctive curved shape resembling a seahorse, consists of the Cornu Ammonis (CA1-3) and the dentate gyrus (DG). Based on neuronal connectivity patterns and detailed gene expression analysis, also confirmed with behavioral studies, it is now accepted that the hippocampus is organized into dorsal and ventral sub regions [16]. This allows dissections of distinct regional functions in this structure. While the dorsal hippocampus (posterior in primates) performs cognitive functions such as pattern separation, spatial memory, and learning [17,18,19], the ventral hippocampus (anterior) is engaged in emotion and mood related functions [20,21,22]. Structural changes to the hippocampus have been suggested to play an important role in the pathophysiology of both neurodegenerative and psychiatric disorders [23]. Another prominent feature of the hippocampus is that adult neurogenesis occurs in the DG. This has recently inspired further studies in this field which have the potential of addressing the role of the hippocampus as a model brain structure for many neurological disorders. 

### 2.2. Hippocampal Neurogenesis

Adult neurogenesis occurs in the mammalian brain, including in humans, in at least two specific anatomical regions, namely the subventricular zone (SVZ) of the lateral ventricles and the subgranular zone (SGZ) of the dentate gyrus in the hippocampus. The SGZ harbors neural stem cells (NSCs), a population of radial glia-like cells (type 1 cells). They give rise to neural progenitor cells (NPCs) which first show glial (type 2a) and then neuronal (type 2b) phenotypes. In the next stage, NPCs become neuroblasts (type 3) that slowly enter a post-mitotic, maturation stage where they gradually move into the granule cell layer and differentiate into neurons (granule cells) [24,25]. These newly formed granule cells, over the course of four weeks, form dendritic trees that become innervated by axons of the perforant path, and they form axons that connect to CA3 pyramidal cells [26,27]. Numerous studies in rodents have unequivocally described a role for new DG neurons in memory, pattern separation, mood, and reward [28,29,30]. Neurogenesis modulation has been attributed to various intrinsic as well as extrinsic factors orchestrated via several signaling pathways including Notch [31,32], Wnt [33], and Sonic Hedgehog (Shh) [34]. A significant modulator of hippocampal neurogenesis is inflammation, which is known to have the greatest effect on neuropathology of neurodegenerative and psychiatric disorders. Additionally, chronic pain is being increasingly associated with anxiety, depression, and deficits in learning and memory, and recent studies highlight abnormalities in hippocampal function and apparent decreases in neurogenesis in patients and rodent models during chronic pain [35]. For simplicity, in the remaining text neurogenesis will always refer to adult hippocampal neurogenesis.

## 3. Inflammation

Neuroinflammation can be initiated in response to various cues such as infection, traumatic brain injury, and autoimmunity. The central nervous system (CNS) has been considered an immunologically privileged site since it is distinctly separated from the peripheral immune system by the blood-brain barrier (BBB) [36]. However, it has emerged that immune B and T cells can infiltrate into the CNS from the periphery and mediate protracted but specific immune responses. On the other hand, the CNS has its own primary mediators; microglia reside within the CNS, providing rapid response to infection or injury and therefore generally performing many of the immune system related tasks.

Microglia in their quiescent or ramified states are responsible for the maintenance of brain homeostasis under normal conditions. In the hippocampus, resting microglia actively participate in neurogenesis through their phagocytic actions [37]. However, under pathological conditions they undergo complex morphological and functional alterations upon activation. Depending on their mode of activation, microglia can have either pro-inflammatory (classically activated) or neuroprotective (alternatively activated) effects [38]. Pro-inflammatory activation is characterized by the release of several pro-inflammatory and neurotoxic factors including nitric oxide, tumor necrosis factor (TNF)-α, interleukin (IL)-6, IL-1β, and IL-12. Polarization towards classic activation can be induced experimentally by exposure to pro-inflammatory cytokines such as TNF-α, interferon (IFN)-γ, IL-1, or bacterial-derived lipopolysaccharide (LPS). M1 cells are competent in antigen processing and presentation [39] and are promoters of cytotoxicity and inflammation [40]. Anti-inflammatory activation of microglia is characterized by increased expression of the anti-inflammatory cytokines IL-4 and IL-10 and factors like transforming growth factor (TGF)-β, insulin-like growth factor (IGF)-1, nerve growth factor (NGF)-1, and brain-derived neurotrophic factor (BDNF). Alternative activation can be induced by application of IL-3 or IL-4. However, in injured or infected brain, the regulation of these activation processes is not so clear-cut and is rather a dynamic process [41].

### 3.1. Inflammation and Neurogenesis

In recent years, it has become evident that hippocampal neurogenesis is significantly affected by microglia. In the absence of inflammation, ramified microglia are involved in providing trophic support for newly forming cells [42]. Microglia also have a phagocytic function, as shown in a stroke mouse model where they are able to remove endothelial cells [43]; in a similar manner, they can mediate apoptosis of newborn cells that are otherwise destined to integrate into the preexisting circuitry in the hippocampus [37]. Upon inflammation, classically activated microglia produce pro-inflammatory cytokines IL-1β, IL-6, and TNF-α, all of which are primarily negative inhibitors of proliferation and cell fate of NSCs in the hippocampus.

In transgenic mice overexpressing astrocytic IL-6 in the hippocampus, reduced proliferation, survival, and neuronal differentiation has been demonstrated [44,45]. Additionally, it was shown in rats that nuclear factor-κB (NF-κB) mediated IL-6 induced depression-like behaviors and decreased the proliferation of hippocampal cells [46]. Another recent study found that when adult rat hippocampal NSCs were cultured in vitro with IL-6 for up to seven days, the number of differentiated neurons and the length of their neurites were significantly increased [47]. It appears that IL-6 might have context-dependent effects on neurogenesis subject to the duration of exposure of NSCs to IL-6, for example. Extensive data demonstrates the negative regulation of neurogenesis by IL-1β. IL-1 receptor knock-out mice did not display impaired neurogenesis, suggesting that this process is critically dependent on IL-1 signaling, while elevated levels of brain IL-1 were associated with various aspects of depression, including the behavioral symptomatology and reduced neurogenesis [48,49]. IL-1β is also involved in the IFN-γ-induced suppression of neurogenesis [50]. Moreover, it was reported that a kynurenine 3-monooxygenase inhibitor reversed IL-1β-induced impaired neurogenesis, suggesting that IL-1β regulates hippocampal neurogenesis via the kynurenine pathway [51]. Similarly, TNF-α, another microglial secreted factor, has been described in in vitro experiments to exert anti-neurogenic effects in the hippocampus [52]. However, TNF functions via two distinct receptors which often mediate opposing biological functions: the pro-inflammatory TNF receptor 1 (TNFR1) and the likely neuroprotective TNF receptor 2 (TNFR2) [53]. Interestingly, TNF has been proven to have a key role in the development of neuropathic pain [54], which will be discussed later. Both TNFR1 and 2 were found to be expressed by hippocampal progenitors and a negative role of TNFR1 in neural progenitor proliferation was identified [55]. Upon inflammation associated with status epilepticus (SE), TNF-R1^−/−^ and TNF-R1/R2^−/−^ mice produced more new neurons and had elevated cell proliferation, while TNF-R2^−/−^ mice showed reduced SE-induced neurogenesis [55,56].

Furthermore, microglia can acquire an alternative neuroprotective phenotype which plays a role in regenerative processes. Evidence suggests that anti-inflammatory cytokines may support neurogenesis. Batista et al. found that TGF-β is involved in increased hippocampal neurogenesis following adrenalectomy. They showed that inhibition of TGF-β with a blocking antibody reduced neuronal differentiation indicating that TGF-β supports newly proliferated cells on their way to becoming neurons [57]. It was also shown that the co-culture of NPCs with microglia stimulated with IL-4 resulted in increased numbers of doublecortin (DCX)-positive cells (immature neurons) compared to non-stimulated microglia [58]. Additionally, stimulating microglia with IL-4 increased microglial expression of IGF-1, which is known to support neurogenesis [59]. Another cytokine, IL-10, was also found to have a positive effect on neuronal differentiation and new cell survival [60].

In addition, increased neurogenesis was achieved when aged rats were treated with fractalkine, a neuroimmune regulatory molecule released by neurons that acts on CX3CR1, receptors expressed by microglia [61]. This ability of fractalkine to increase the number of newborn DG neurons is thought to result from reductions in IL-1β mRNA levels. However, mice with CX3CR1 deletion exhibit resilience to chronic stress. Unlike CX3CR1 knock-out mice, wild-type mice following stress showed reduced sucrose preference, impaired novel object recognition memory, and reduced neurogenesis [62]. These findings suggest inhibition of CX3CR1 signaling as a novel approach for promoting stress resilience. 

Balancing the ratio of pro- and anti-inflammatory cytokines could be one of the resilience mechanisms; when the proportion of anti-inflammatory cytokines prevails, resilience is achieved and if pro-inflammatory cytokines prevail, vulnerability is increased.

### 3.2. Inflammation and Neurological Disorders

The main functions of the hippocampus, cognition and emotion, become impaired in many neuropsychiatric diseases. Despite the diverse and specific causes of each disease phenotype, a common hallmark for the symptoms includes memory deregulations and mood disorders.

Inflammation caused by macrophages was reported to possibly play a crucial role in the pathophysiology of depression in 1991 [63]. Subsequently, a close association between pro-inflammatory alterations and depression has been reported [64]. Furthermore, increased neuroinflammation was associated with bipolar disorder [65]. A vast amount of literature describing inflammation using animal models emphasizes the causal role of inflammatory signaling in memory and cognitive deficits (reviewed in [66]). Specifically, studies performed in mice have determined that the deletion or inducible silencing of new DG neurons impairs aspects of memory function [67,68] either in the memory acquisition or memory retrieval [69,70]. These studies, all of which manipulate adult neurons during or before learning, suggest that enhancing neurogenesis may improve memory. In another approach, systemic injection of LPS, a common method used for experimentally mimicking inflammation, has shown that its application leads to impaired memory consolidation [45] and learning in Morris Water Maze tasks long-term potentiation (LTP) [71]. Pro-inflammatory cytokines have been associated with memory dysregulations. Elevated hippocampal IL-1β levels produced marked impairments in spatial memory tested with the water maze paradigm, as well as impaired long-term contextual fear memory [72]. Additionally, TNF-α has been consistently implicated in deficits of memory and plasticity. Overexpression of TNF-α in neurons or glial cells impairs passive avoidance memory and synaptic plasticity [73], while IL-6 overexpression causes broad memory impairments and diminished LTP [74].

A pro-inflammatory state has been associated with mental disorders, including major depression, anxiety, and schizophrenia. Increased inflammation has been suggested as a risk factor for developing a mood or psychotic disorder [75]; anti-inflammatory medication reduces symptoms of depression in antidepressant-resistant populations [76]. Activating inflammation in the brain of rodents induces behavioral phenotypes of depression, anxiety, and schizophrenia in correlation with reduced proliferation and survival of new neurons in the DG [77]. Further evidence for correlation between fewer new DG neurons and the presence of neuropsychiatric symptoms derives from studies in both humans and rodents in which a decreased number of new neurons related to lower DG volume is normalized or improved after treatment or during remission of the disease [78]. 

## 4. Pain

Another emerging field of study in the context of neurogenesis is chronic pain. Pain is an unpleasant sensory and emotional experience that is associated with actual or potential tissue damage. In general, pain is an important defense mechanism. However, prolonged pain can result in processes that may cause severe damage.

Chronic pain is defined as any pain lasting more than 12 weeks and is an aberrant somatosensory processing in the periphery or CNS. Chronic pain is generally classified by physiological changes that are associated with the injury or illness as nociceptive (due to persistent tissue injury), neuropathic (due to damage to the brain or spinal cord), or visceral (due to nociceptor activation in the internal organs). Neuropathic pain, defined as “pain caused by a lesion or disease of the somatosensory nervous system” [79], is a specific type of chronic pain that results from damage of the neurons during injury to the periphery or CNS, and does not primarily signal noxious tissue stimulation. Chronic pain is a complex process, associated with abnormal mood and memory, therefore it is expected that hippocampal processes are involved. In line with this, recent evidence demonstrates impairments in hippocampal function, changes in associated behavior, and changes in hippocampal neurogenesis.

### 4.1. Pain and Neurogenesis

The hippocampus is one of the brain regions that play a key role in modulating pain signals; it is activated during pain processing and modification of nociceptive stimuli. Chronic pain interferes with hippocampal mossy fiber-CA3 synaptic plasticity and DG neurogenesis (shown in Table 1), which is still being explored in this field. Alterations of these hippocampal properties may relate to hippocampal volume loss seen in chronic pain patients [80]. It was shown that older adults who display severe acute pain or chronic pain have smaller hippocampal volumes and lower levels of hippocampal NAA/Cr, a marker of neuronal integrity and neuronal loss. Studies on chronic pain in human and animal models have shown hippocampal volume loss and pathophysiological changes in the hippocampus, which can give an estimation of behavioral manifestations of anxiety and increased susceptibility to stress [81]. Interestingly, resilient animals have even exhibited an increase in hippocampal volume (by 4%), even after stress [82]. Using mouse models of neurogenesis via specific morphogens like NSE (neuron specific enolase)-Noggin and NSE-BMP4, and X-irradiation for ablating neurogenesis followed by examination of pain as a result of inflammatory or neuropathic peripheral injury indicates decreased neurogenesis. This in turn leads to complete blockade or delayed and decreased post-injury pain behavior [35]. When the rate of neurogenesis was quantified by counting bromodeoxyuridine (BrdU)-positive cells in the hippocampal DG 24 h after the administration of BrdU, results demonstrated that exposure to either acute nociception (formalin) or acute stress (a single 45-min immobilization) did not alter the number of BrdU-labeled cells relative to the controls [83]. However, when animals were exposed to either prolonged nociception (via application of Complete Freund’s Adjuvant (CFA) for 21 days in order to achieve hyperalgesia) or stress (10 days of repeated immobilization) the number of BrdU-positive cells in the DG was significantly decreased. A commonly used drug to treat neuropathic pain and anxiety, pregabalin, decreases neuronal excitability of DG neurons and accelerates maturation of new-born neurons in vivo, suggesting that these actions enable its anxiolytic properties [84]. Moreover, axonal regrowth was accelerated and neuromuscular synapses restored after injury of the sciatic nerve upon progranulin overexpression, leading to recovery of sensory and motor function [85].

Furthermore, chronic pain was explored in the context of environmental enrichment (EE), which is known to induce hippocampal neurogenesis [86]. Upon EE, there was a significant increase in the DCX- and NeuroD-positive cells in DG, however, this effect was abolished in the presence of chronic pain. These results suggest that chronic pain has stress-like damaging modulatory effects on hippocampal neurogenesis [87]. Additionally, TNF-α, a pro-inflammatory cytokine, was found to also play a role in neuropathic pain and is associated with impairments in hippocampal neurogenesis. Neuropathic pain resulted in the development of depressive symptoms in a time dependent manner and was associated with impaired neurogenesis, as well as reduced expression of neuroplasticity markers and myelin proteins. The onset of depressive-like behavior correlated with increased hippocampal levels of TNF, and decreased expression of TNFR2, which were all fully restored after mice recovered from pain. Notably, TNFR1^−/−^ mice did not develop depressive-like symptoms after injury, nor were there changes in hippocampal neurogenesis and plasticity [88].

Neurogenesis occurs in the human brain as well [89] and it would be of great importance to understand its effects in patients with chronic pain. However, there are few studies available regarding this topic, partially due to technical limitations. What has been reported is that patients with chronic back pain have smaller hippocampi and stronger phasic pain responses in the bilateral anterior parahippocampal gyrus [90]. One of the main findings of this study is that the level of basal cortisol and the clinical pain intensity of patients with chronic back pain are associated with increased pain-related responses in the anterior hippocampal formation. Additionally, reduced hippocampal volumes have been reported in chronic back pain patients and complex regional pain syndrome patients, while this effect was absent in knee osteoarthritis patients, possibly due to the type of pain [80].

When it comes to emotional aspects of resilience to pain, a key approach that helps individuals resist the damaging effects of pain is maintaining high average levels of positive emotion, usually measured as elevations in positive affect. Positive affect has been associated with a wide range of benefits: it has been linked to lower negative affect and pain over the course of time [92], and those who have higher average levels of positive affect have been found to have more responsive immune systems [93]. Concerning cognitive-behavioral aspects, one of the prominent roles in resilience to pain belongs to active coping. Active coping refers to directed actions by an individual in pain to control their own pain and to function in spite of any pain that they are experiencing [94]. Active coping has been associated with improved physical activity levels [94], higher levels of social interaction [95], and lower levels of depression [96].

### 4.2. Pain and Neurological Disorders

Cognition, the brain’s ability to acquire, process, store, and retrieve information, is negatively affected in chronic pain conditions [97]. Chronic pain is often a symptom of complex disorders such as migraine headaches, irritable bowel syndrome, and diabetes, which also express comorbid depression. Therefore, it is not surprising that chronic pain, depression, and cognition display overlap in the areas of attention, learning, and memory, which are hallmarks of hippocampus function. In particular, LTP, a type of synaptic plasticity associated with learning and memory formation, is impaired in hippocampal slices from nerve-injured mice [98]. Furthermore, the nerve injury model of neuropathic pain in rodents presents increased TNF-α production in the hippocampus leading to memory impairment and hippocampal dysfunction [99]. Cognitive impairment is further demonstrated in transgenic mice that overexpress TNF-α. Interestingly, animals with increased TNF-α are susceptible to developing chronic pain and depressive behaviors [99]. These findings point toward a common potential mechanism contributing to the emotional component of pain and its impact on cognitive functioning. Moreover, attenuated BDNF release in the hippocampus followed by decreased early immediate gene c-Fos levels and neurogenesis, is associated with persistent pain and mental deficits observed after orthopedic surgery [100]. On the contrary, elevation of BDNF by diverse treatments ranging from antidepressant drugs, such as fluoxetine, to regular physical activity may be a key feature of treatment [101] or a mechanism of resilience.

Chronic pain patients appear to suffer as much from the emotional disturbance as from the pain itself, supporting the role of the hippocampus in the processing of chronic pain. Comorbidities such as fatigue, anxiety, and depression, as often observed in neuropathic pain patients, could be justified with the emotional component of pain [102]. In patients with chronic pain, more than 50% suffer additionally from a type of depressive disorder. The link between chronic pain and depression is often labeled by clinicians as a depression-pain syndrome [103], which implies the often occurring coexistence. However, it is not yet clear which of the two conditions occurs first and whether there is a causal link between them. 

## 5. Conclusions

**Figure 1:**
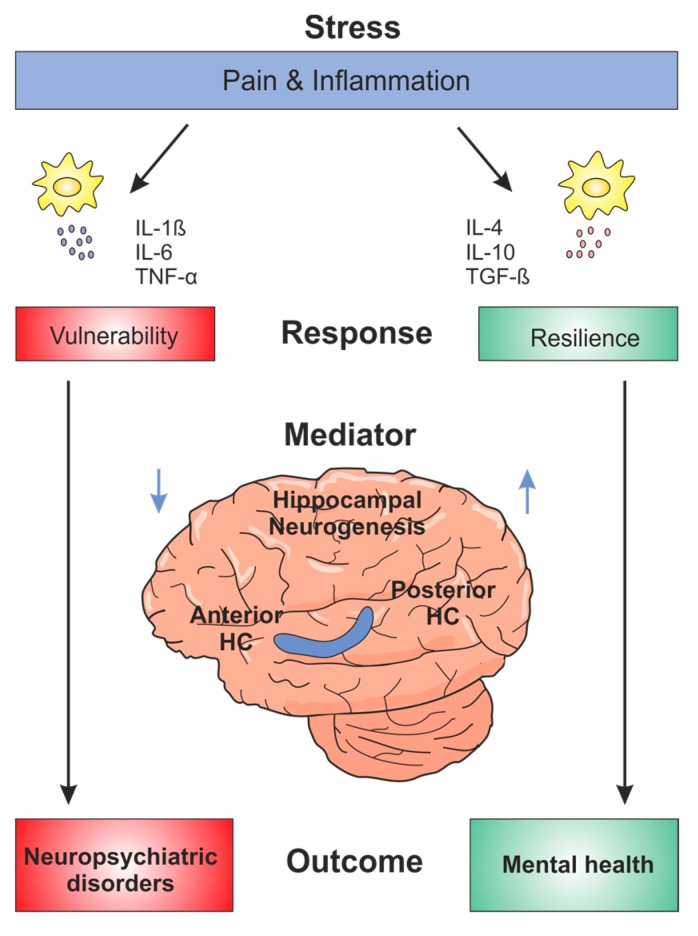
Resilience and vulnerability of the human hippocampus. Hippocampal neurogenesis can be modulated positively and negatively, as indicated with blue arrows. Various factors, such as stress or chronic pain, can negatively regulate neurogenesis and further cause dysregulations of emotional and cognitive functions associated with neuropsychiatric disorders. On the contrary, individuals can employ mechanisms of resilience and maintain homeostasis even under negative conditions. Alternatively, neurogenesis can be modulated in a positive manner and used as a therapeutic means. TNF: tumor necrosis factor; IL: interleukin; TGF: transforming growth factor; HC: hippocampus.

In recent years, the major focus of adult neurogenesis research has been to examine the function of newborn neurons in the hippocampus and their role in neurological and psychiatric diseases (summarized in Figure 1). Decreased hippocampal volume and impaired neurogenesis have been reported for major depression, bipolar disorder, schizophrenia, and addiction. The progress achieved in characterizing dysfunctions during human neurogenesis reveals that enhancing neurogenesis represents a powerful tool for modulating memory and mood dysregulations associated with chronic pain and inflammation conditions. So far, the main research focus has been how to undo the negative effects that lead to disease, but recently a new field of research has emerged providing a novel approach for treatment by finding ways to induce resilience. Based on data presented in this review, it can be concluded that suppression of adult neurogenesis enhances vulnerability of the hippocampus, under conditions of pain and inflammation. On the contrary, enhancing neurogenesis can be seen as a resilient mechanism and moreover, adult neurogenesis could contribute to resilience by regulating the processing of both cognitive and emotional information. Therefore, it is important to study and investigate both functions of the hippocampus, memory and mood, in close correlation, given that both of these functions are deregulated in neuropsychiatric diseases. The link between pain and neurogenesis has not been researched extensively and resilience to pain mostly includes psychological and social approaches. What we propose here is that neurogenesis could contribute to pain resilience by regulating emotional and cognitive hippocampal functions. It has already been shown that running and other forms of exercise increase neurogenesis [104,105,106]. Furthermore, antidepressant treatment (also used to treat pain [107,108]) has been shown to increase neurogenesis; the effects of antidepressant drugs are proposed to be mediated via routine pathways activated during neurogenesis [28]. In addition, it is possible that antidepressants exert their effects by inducing some of the same adaptations that occur in resilient individuals [109].

## Figures and Tables

**Table 1:** Pain and neurogenesis.

Pain	Neurogenesis	Effect on Neurogenesis	Publication
Neuropathic pain	↓	Suppressed neurogenesis	[91]
Persistent pain; spared nerve injury	↓	Reduced BrdU/DCX cells	[80]
Persistent pain; spared nerve injury	↑	Neurogenesis mouse models	[35]
Persistent pain	↓	Reduced BrdU cells	[83]
Neuropathic pain	↓	Reduced NeuroD cells	[87]
Neuropathic pain	↓	Reduced BrdU/NeuN cells	[88]

BrdU: bromodeoxyuridine; DCX: doublecortin; NeuN: neuronal nuclear antigen; NeuroD: neurogenic differentiation factor.

## Abbreviations

CA1-3	Cornu Ammonis
DG	Dentate gyrus
SVZ	Subventricular zone
SGZ	Subgranular zone
NSCs	Neural stem cells
NPCs	Neural progenitor cells
CNS	Central nervous system
TNF	Tumor necrosis factor
IL	Interleukin
TGF-β	Transforming growth factor-β
IGF-1	Insulin-like growth factor
NGF-1	Nerve growth factor
BDNF	Brain-derived neurotrophic factor
LTP	Long-term potentiation
LPS	Lipopolysaccharide
SE	Status epilepticus
